# Evaluating the Effectiveness of Primary Care Health Checks at Assessing Cardiovascular Risks among Ethnic Minorities in the UK: A Systematic Review

**DOI:** 10.31083/RCM25614

**Published:** 2025-01-21

**Authors:** Aleesha Karia, Reza Zamani, Tanimola Martins, Abdal Zafar, Ava Zamani

**Affiliations:** ^1^Medical School, Faculty of Health and Life Sciences, University of Exeter, EX1 2LU Exeter, UK; ^2^Department of Trauma and Orthopaedics, The Royal London Hospital, E1 1FR London, UK; ^3^Department of Medical Oncology, St Bartholomew’s Hospital, EC1A 7BE London, UK

**Keywords:** cardiovascular disease, NHS health check, ethnic minorities, primary care, prevention

## Abstract

**Background:**

Cardiovascular diseases (CVD) affect around 7.6 million people in the UK, disproportionately affecting the minority ethnic community. In 2009, the UK's National Health Service (NHS) launched a Health Check (NHSHC) scheme to improve early diagnosis of various clinical conditions, including CVD, by screening patients for associated risk factors. This systematic review investigated the engagement of minority ethnic groups with these services.

**Methods:**

Seven studies identified patient demographics of NHSHC attendees using the Preferred Reporting Items for Systematic And Meta Analysis-Diagnostic Test Accuracy (PRISMA-DTA) guidelines and accessing Ovid (MEDLINE), PubMed and Web of Science databases.

**Results:**

The screening was either by invitation or opportunistic at other appointments with their doctor. Engagement with the service was highest among the South Asian patients (21%–68%), but lowest amongst Chinese patients (12%–61%). Further, engagement was lower among those screened following a formal invitation than those seen opportunistically. However, a greater proportion of patients were screened opportunistically than by invitation.

**Conclusions:**

Overall, we found that the NHSHC is not being utilised adequately for all patients at high risk of CVD, particularly White and Chinese patients. It highlights the critical role of primary care could play to improve patient engagement with the service.

## 1. Introduction 

Cardiovascular diseases (CVD) affect around 7.6 million people, with over 170,000 deaths reported each year in the UK. Primary care plays a critical role in chronic disease prevention and management and serves as the gatekeeper to secondary and specialist services in the UK [1]. Typically, when a patient presents with suspected CVD symptoms in primary care, the general practitioner (GP) will often conduct initial assessments to determine whether a specialist investigation and associated referral is warranted [2]. However, there are sociodemographic variations in access to and experiences of care in the UK, including primary and specialist care [3]. In particular, UK ethnic minorities (including the British Black and Asian groups) bears disproportionate burden of CVD risk factors [4, 5], such as high body mass index (BMI), diabetes, and hypercholesterolemia [6]. These groups are also more likely to be diagnosed with CVD at secondary care, which may be indicative of suboptimal primary care and is associated with advanced-stage disease at diagnosis [5]. Research attributes part of this to patient-related factors, including socioeconomic deprivation and poor knowledge of navigating UK healthcare, alongside healthcare system-related factors, including difficulty in booking GP appointments and perceived racial discrimination within healthcare [7].

Recognition of early warning signs and CVD risk factors could reduce the chances of heart failure and episodes of myocardial infarction [8]. Evidence suggests that patients often misattribute early warning signs or delay seeking medical care, thus developing advanced-stage disease, which often requires radical treatment [9]. The overall management of CVD and associated conditions cost the UK an estimated £28 billion per annum [10]. Preventative intervention may reduce the chances of disease progression and reduce costs in secondary care.

To facilitate early detection of CVD, the National Health Service (NHS) introduced the CVD Health Check (NHSHC) scheme in 2009 [11], allowing asymptomatic patients registered with a GP to be assessed for CVD risks and referred to specialist services [12]. Although this service was designed as an early intervention, there is little evidence of its impact on improving access to CVD services among ethnic minorities. The NHS Long-term Plan to improve CVD outcomes include a commitment to tackle inequalities in health through this service [13].

In the present study, we aim to critically examine the evidence regarding the barriers to accessing primary care services for patients at risk of CVD. Specifically, we investigated existing literature to assess ethnic differences in patients’ engagement with the NHSHCs for CVD risk assessment in primary care.

## 2. Methodology 

The systematic review followed the Preferred Reporting Items for Systematic And Meta Analysis-Diagnostic Test Accuracy (PRISMA-DTA) guidelines for systematic reviews [14]. The Population, Intervention, Context and Outcome (PICO) framework was used to develop the question and search strategy (Table 1).

**Table 1.** **The PICO (Population, Intervention, Context and Outcome) format used for the literature search**.

Population (P)	Intervention (I)	Context-control and Outcome (CO)
Terms relating to ethnicities of patients at risk of CVD risk	Terms relating to CVD risk screening or assessment, e.g., NHSHC in the UK primary care system	Terms relating to access, attendance or engagement with the intervention

NHSHC, National Health Service Health Check; CVD, cardiovascular diseases.

### 2.1 Database Search

Ovid MEDLINE, PubMed and Web of Science databases were searched between the 13th of November 2022 and the 10th of September 2023 using search terms: Prevention, Cardiovascular Disease, Ethnicity, Primary care, General Practice, Community pharmacy, Family practice, and Family doctor. Specific search terms were selected to highlight any intervention (treatment, diagnostics or monitoring of symptoms or risk factors assessment) associated with CVD. The term ‘prevention’ aims to identify studies investigating interventions for screening CVD risk factors in primary care. Primary care was defined as all interventions offered by the GP, nurse, or other allied health professionals in primary care and community settings. Therefore, the keywords ‘general practice’, ‘community pharmacy’, ‘family practice’ and ‘family doctor’ were added to the search. A detailed search strategy for each database is provided in Appendix 1.

All articles retrieved from the searches were managed using Rayyan Software (https://rayyan.ai/), web tool designed to facilitate studies screening and selection in systematic reviews. After removing the duplicates, AK (benchmarked against with RZ, TM and AZaf) manually screened studies based on the eligibility criteria.

### 2.2 Eligibility Criteria 

Eligible studies were those conducted in the UK and included patients aged at least 40 years at enrolment in the study, with no previous diagnosis of CVD or related risk factors. They included studies published between 2012 and 2022, compared at least two different ethnic groups and presented relevant data on CVD risk factors or inequalities in the need for, or access to CVD screening. Studies exploring CVD treatment or management of patients with terminal diseases were excluded. Other exclusions were conference abstracts, with no available full text, and studies that focused on non-UK cohorts. However, no study was excluded based on design, sample size or quality.

### 2.3 Study Selection 

Considering the trends in the NHSHC uptake [15, 16, 17, 18], there has been a steady increase in the number of patients attending appointments.

The initial screening process excluded titles and abstracts of studies with no data reflecting primary care services within the UK and CVD or CVD risk factors. The remaining papers were screened in full text, and the data presented was compared with the eligibility criteria. Two independent reviewers (AK and AZaf) screened papers, then unblinded to discuss conflicting reports.

### 2.4 Data Extraction and Synthesis

Patient demographics (ethnicity, age, biological sex, and a measure of deprivation) were extracted from the data available. Ethnicity was cumulatively compared with the UK 2011 census, as this is the closest to the eligible studies data collection period [19]. The ratio of each ethnic group within our review was compared with the ratio of the same ethnicity residing in the UK population (2011 census) and presented as a percentage (called degree of representation—Table 2, Ref. [15, 16, 17, 18, 19, 20, 21, 22, 23, 24]).

**Table 2.** **Socioeconomic demographics of the final selection of studies**.

Study citation		Robson *et al*. [15]	Garriga *et al*. [20]	Patel *et al*. [16]	Gulliford *et al*. [21]	Chang *et al*. [17]	Robson *et al*. [18]	Total	Degree of representation (%)*
Ethnicity									
	South Asian	42,770	30,382	261,431	360	654	4993	340,590	93
	African Caribbean	31,036	20,740	148,160	1426	424	4583	206,369	89
	White	733,851	481,204	4,067,864	1336	14,562	9935	5,308,752	94
	Chinese	5295	3639	27,360	-	-	-	36,294	77
	Other	35,369	15,760	221,975	1871	283	1445	276,703	87
	Missing	42,872	29,736	375,968	366	4486	238	453,666	
Age									
	40–49	419,149	286,559	1,951,264	-	7584	-	2,664,556	
	50–59	265,898	177,627	1,742,003	4583	6841	18,056	2,215,008	
	60+	206,146	126,032	1,409,491	776	5984	3138	1,751,567	
Sex									
	Male	402,129	260,748	2,311,604	2478	9250	-	2,986,209	
	Female	489,064	329,470	2,791,130	2881	11,159	-	3,623,704	
Deprivation		Townsend	Townsend	IMD	IMD	IMD			
	Least deprived	203,569	133,493	1,129,670	1723	3903	-	1,472,358	
		193,417	131,539	1,094,925	2843	4267	-	1,426,991	
		174,218	118,238	1,027,096	525	4023	-	1,324,100	
		156,090	103,569	954,656	17	4457	-	1,218,789	
	Most deprived	163,151	102,841	893,194	-	3759	-	1,162,945	

*= Degree of representation is based on the 2011 UK census statistics [19]. Where there are missing data points, data was not available to us or grouped into other categories. Missing data for ‘Chinese participants’ have been categorised as ‘Other Asian’ in three studies; this was collected in “other” for our study [17, 18, 21]. Woringer* et al*.’s [22] data could not be collated in this table as raw data was not available to us. The table collates the demographics of patients attending their NHSHC. This is reflected by the engagement of patients with the service. All people aged between 40–74 are eligible for an NHSHC at their local providers. Two studies [18, 21] pooled the data in age categories of 40–59. Deprivation was either measured as a Townsend score or Index of Multiple Deprivation (IMD) [23, 24]. NHSHC, National Health Service Health Check.

### 2.5 Risk of Bias 

The Newcastle Ottawa Scale for cohort studies was adapted to assess the risk of bias for each paper (Appendix 2) [25]. This evaluated the representativeness of study participants’, selection of non-exposed cohort, ascertainment of exposure, compatibility, and assessment of outcome. Given the evidence of regional variation in care [3], we assessed the representation of cohorts on how well they reflect the population accessing the service in the region where the data is collected. Studies based on electronic health records of service users were considered to be representative of the target population. Based on the Newcastle Ottawa scale, a study can achieve a maximum of 2 stars for its data collection method if it is from two sources (e.g., self-reported data or electronic records), which allows for different methods of collecting demographic and clinical data. We have adapted the scale to award “self-reported” ethnicity data with a star, understanding that ethnicity is a self-reported value. We also awarded a star where studies have adjusted for potential confounders (e.g., age, sex and marital status) in the analysis.

## 3. Results 

### 3.1 Search Results 

**Fig. 1.**
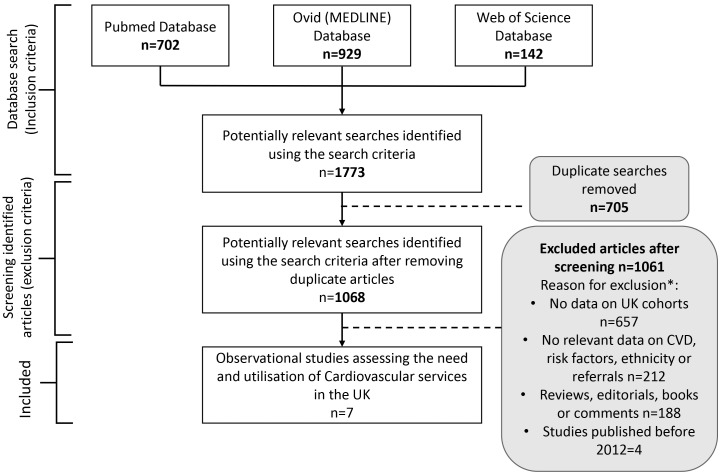
**Flowchart: Preferred Reporting Items for Systematic and Meta Analysis (PRISMA) Flow chart of studies systematically reviewed for this study**. The flowchart shows the selection of papers following the inclusion and exclusion criteria of the systematic review. Dashed lines show where articles were excluded, and arrows represent the papers screened further. * Reasons for exclusion may be multifactorial, but only one reason was recorded for exclusion (e.g., an article may use cohort outside of the UK and not include data on cardiovascular disease (CVD)).

The search returned 1773 potentially relevant papers (Fig. 1). Of these, 705 were duplicate results. Once removed, abstract and title of 1068 papers were screened based on the eligibility criteria, and 1040 ineligible studies were subsequently removed. The reasons for exclusion are highlighted in the PRISMA Flowchart (Fig. 1). The remaining 28 papers were screened for full-text review. Upon further discussion, articles were excluded as they did not focus on the patient experience and engagement with CVD risk assessment service [26, 27]. At this stage, a study by Tillin* et al*. [28], was excluded due to a lack of data regarding access to the service despite discussing the impacts of the health check on CVD outcomes by ethnicity.

Of the studies collected, seven that recorded patient engagement with the NHSHC were included in the study for final analysis. the seven studies comprised six cohort and one cross-sectional study.

### 3.2 Study Characteristics

The characteristics of the seven papers eligible for the review are presented in Table 3 (Ref. [15, 16, 17, 18, 20, 21, 22]). The seven studies comprised 6,622,374 patients, 80.2% of whom were white, with 3.1% being Black, 5.1% South Asian, 0.5% being Chinese, 4.2% categorised as other, and 6.9% with missing ethnicity data. 40% of the patients were aged 40–49 year, 33.4% were aged 50–59 year and 27% were 60 or over. Around 22% were categorised in the least deprived quartile and 18% in the most deprived quartile (Table 2). Women were slightly over-represented (55%) compared with men (Table 2). Five of the seven studies scored 6/8 on the Newcastle Ottawa scale; the remaining graded 4/8 and 5/8, making them all eligible for analysis (Table 3).

**Table 3.** **Study characteristic of included studies**.

Author	Title	Study design	Location	Year data collected	Attended NHSHC	Healthcare setting	Intervention/aim	Data collection method (incl. secondary data)	Newcastle Ottawa scale	Relevant findings
Robson *et al*. [15]	NHS Health Checks: an observational study of equity and outcomes 2009–2017.	Cohort study	UK	2009–2017	891,193	General practice	Assess uptake of NHSHC and treatment follow-up	QResearch	6	- Increased rates of South Asian attendees compared to all other ethnicities - Type 2 diabetes and hypertension more likely diagnosed in patients of greater deprivation or of South Asian and Black ethnic groups
Garriga *et al*. [20]	NHS Health Checks for people with mental ill-health 2013–2017: an observational study.	Cohort study	England	2013–2017	65,490	General practice	Assess uptake of NHSHC and treatment follow-up in people with serious mental illness and long-term antidepressant medication	QResearch	6	- Non-white ethnic groups more likely to attend NHSHC when compared to White ethnicities, except for Chinese - People living in deprived quartiles less likely to attend NHSHC
Patel *et al*. [16]	Evaluation of the uptake and delivery of the NHS Health Check programme in England, using primary care data from 9.5 million people: a cross-sectional study.	Cross-sectional study	England	2012–2017	5,102,758	General practice	Assess uptake, process and delivery of NHSHC, follow-up treatment and sociodemographic risk factors	General Practice Data Extraction Service (GPES)	6	- Increased rates of South Asian patients attending when compared to white ethnicity - Higher uptake of attendees in South London - Increased uptake of NHSHC in more affluent deciles - Lack of evidence to suggest inequality in invitation hand out
Gulliford* et al*. [21]	Cardiovascular risk at health checks performed opportunistically or following an invitation letter. Cohort study.	Cohort study	London	2013–2015	6184	General practice	Compare NHSHC uptake for those invited through the routine system and opportunistic risk assessment	Electronic health records	6	- Higher odds of receiving a >10% CVD risk score in opportunistic NHSHCs in Black, Asian and Mixed ethnicities -Higher odds of receiving a >10% CVD risk score in opportunistic NHSHCs in the most deprived quartiles - More opportunistic NHSHCs in deprived areas
Chang *et al*. [17]	Coverage of a national cardiovascular risk assessment and management programme (NHS Health Check): Retrospective database study.	Cohort study	England	2009–2013	95,571	General practice	Evaluate national implementation of NHSHC and assess the risk factors of attendees	Clinical Practice Research Datalink (CPRD)	6	- Lower attendance rates in Black and Chinese ethnicities for the NHSHC - Variation in coverage of the NHSHC across regions of England and between individual General practices - Coverage of the NHSHC program similar in affluent and deprived groups
Robson *et al*. [18]	The NHS Health Check programme: implementation in east London 2009–2011.	Cohort study	East London	2009–2011	50,651	General practice	Describe implementation of NHSHC and management of new comorbidities of attendees	Electronic health records	4	- Improved coverage of NHSHC intervention over three years -Variation in coverage, finance and practice between GP practices - Variation in financial incentives for different GP practices taking appointments - No significant differences in ethnicities attending NHSHC
Woringer *et al*. [22]	Evaluation of community provision of a preventive cardiovascular programme - the National Health Service Health Check in reaching the under-served groups by primary care in England: cross sectional observational study.	Cross-sectional study	England	2008–2013	43,177	Local community providers	Investigate if engagement with NHSHC would increase with community providers	Health Options software	5	- Community health checks at places other than the local GP more convenient (time and language) - More engagement with the younger population in the north of England by community providers - Increased uptake in Asian communities when compared to the general population - Less representation of the White population attendees compared to the general population - Leicester, Thurrock, Sutton, South Tyneside, Portsmouth and Gateshead more successful recruiting ethnic minority patients when compared to local demographics

This table highlights the main findings of the studies that were used in our systematic review. All studies met out inclusion criteria. Studies collectively investigate attendance of NHS Health Checks (NHSHCs) from 2009–2017. The Newcastle Ottawa Scale was adapted to assess the bias of the studies included [15, 16, 17, 18, 20, 21, 22]. CVD, cardiovascular disease; GP, general practitioner; NHS, National Health Service.

### 3.3 Patient Engagement with the NHSHC 

Five papers discussed the ratio of participants who attended the NHSHC compared to the population eligible to attend [15, 16, 17, 18, 22], one addressed the proportion of participants attending opportunistically compared to being invited [21]. Opportunistic health checks refer to the health checks performed at doctors’ appointments that are not CVD-related. Three studies described the number of eligible populations attending the NHSHC [15, 17, 18], and another study investigated the attendance of people who had received a formal invitation from their GP [16]. Table 4 (Ref. [15, 16, 17, 18, 29]) shows the percentage of people attending NHSHC compared to the number of those eligible to attend the service.

**Table 4.** **Patients attending NHSHC compared to participants eligible for NHSHC (shown in percentages)**.

Citation		Robson *et al*. [15]			Chang *et al*. [17]			Robson *et al*. [18]			Patel *et al*. [16]		
		Attended (n)	Eligible (N)	Engage % (n/N)	Attended (n)	Eligible (N)	Engage % (n/N)	Attended (n)	Eligible (N)	Engage % (n/N)	Attended (n)	Invited (N)	Engage% (n/N)
Ethnicity													
	South Asian	42,770	199,499	21.44	654	1073	60.95	4993	22,695	22.00	261,431	386,028	67.72
	Black	31,036	178,137	17.42	424	1304	32.52	4583	29,142	15.73	148,160	227,449	65.14
	White	733,851	4,082,242	17.98	14,562	49,654	29.33	9935	62,286	15.95	4,067,864	6,946,824	58.56
	Chinese	5295	33,668	15.73	53	176	30.11	1445	12,491	11.57	27,360	44,730	61.17
	Other	35,369	223,542	15.82	283	1075	26.33	-	-	-	221,975	364,877	60.84
	Unknown/missing	42,872	2,203,108	1.95	4486	42,289	10.61	-	-	-	375,968	1,725,071	21.79
Age													
	40–49	419,149	3,842,145	10.91	7584	44,561	17.02	-	-	-	1,951,264	4,195,179	46.51
	50–59	265,898	1,848,193	14.39	6841	30,494	22.43	18,056	128,921	14.01	1,742,003	3,247,358	53.64
	60+	206,146	1,229,858	16.76	5984	20,516	29.17	3138	15,530	20.21	1,409,491	2,252,442	62.58
Sex													
	Male	402,129	3,486,963	11.53	9250	45,708	20.24	-	-	-	2,311,604	4,724,015	48.93
	Female	489,064	3,433,233	14.24	11,159	49,863	22.38	-	-	-	2,791,130	4,970,906	56.15
Deprivation		Townsend	Townsend		IMD	IMD					IMD	IMD	
	Least Deprived	203,569	1,512,548	13.46	3903	16,229	24.05	-	-	-	1,129,670	2,067,637	54.64
		193,417	1,455,336	13.29	4267	20,001	21.33	-	-	-	1,094,925	2,079,256	52.66
		174,218	1,405,828	12.39	4023	21,152	19.02	-	-	-	1,027,096	1,965,158	52.27
		156,090	1,311,918	11.90	4457	20,953	21.27	-	-	-	954,656	1,825,375	52.30
	Most Deprived	163,151	1,223,255	13.34	3759	17,236	21.81	-	-	-	893,194	1,750,356	51.03

According to the NHS guidelines, the eligible population is people between the ages of 40 and 74 [29]. This table highlights whether the targeted demographic is engaging with the service by attending. One study looks at the attendance of people invited by their service provider [16]. NHSHC, National Health Service Health Check; NHS, National Health Service; IMD, Index of Multiple Deprivation.

All of the five studies comparing the number of eligible patients attending the NHSHC reported a greater percentage of South Asian patients engaging with the services compared to other ethnicities [15, 16, 17, 18, 22]. Two of these also showed that Black patients were more likely to engage with services than White patients [16, 17]. A further two studies found little evidence of a difference in the engagement between White and Black patients [15, 18].

Only three of the seven studies investigated the attendance of Chinese patients at the NHSHC. The studies showed that Chinese patients were less likely than White patients to attend the service [15, 16, 18]. The engagement rates of Chinese patients across the three studies ranged from 12–30%, compared with 16–29% in the White group, 16–33% in Black, and 21–61% in South Asian group.

The study conducted by Patel *et al*. [16], examined the number of attendees in comparison to eligible patients who were formally invited by the GP. They found that formal invitation by the GP led to an increase in engagement across all ethnic groups. For instance, among those formally invited engagement was 61% among Chinese, 65% among Black, and 59% among White patients. These proportions are considerably larger than those reported in the three studies that examined engagement of formally as 61% and 65%, respectively, compared to 59% in White patients.

The study by Gulliford *et al*. [21] investigated the reason for attendance and compared the patients attending in response to an invite to those attending opportunistically. The authors reported that most of the participants, irrespective of ethnicity or other demographic characteristics, were accessing NHSHC opportunistically rather than via GP invitations. They showed that an additional 843 patients were assessed opportunistically, with the Black (n = 324) followed by White patients (n = 270) more likely than other groups to access the NHSHC opportunistically. The corresponding figures were 164 extra Mixed and 74 extra Asian patients.

Gulliford *et al*. [21], also reported that opportunistically-screened patients had higher odds of CVD risk score at their appointment (*p*-value < 0.001) than those formally invited to the service. Comparing those formally invited to those screened opportunistically, the odds of CVD risk was lower in the White group [odds ratio = 1.49; 95% CI: 1.17–1.89] compared with Asian [odds ratio = 1.66; 95% CI: 1.03–2.69] or Black group [odds ratio = 1.74; 95% CI: 1.37–2.21].

## 4. Discussion 

### 4.1 Demographics 

Around 7% of the participants in this review had “missing” or “not reported” ethnicities, similar to the 10% of missing ethnicity data found in UK electronic health records [30]. Given the strong association between ethnicity and CVD risk [4, 5, 31], it is important to understand the reasons behind the missingness and how these can be mitigated. A full knowledge of patients’ ethnicity will not only deepen our understanding of broader risk factors for each ethnicity but will help clinicians with developing a targeted response for each community for engagement and eventual treatment of CVD. Between 2006 and 2011, the UK Quality and Outcomes framework recognised the importance of ethnicity data and offered GPs a financial incentive to increase the recording of this variable [30].

There are several reasons why ethnicity data collection may be suboptimal in a clinical setting. For example, staffing pressures, lack of capacity or willingness on patients’ part to self-report ethnicity. Although this may be the case for a minority of patients or healthcare settings, it does not justify the proportion of missing data in this study and challenges the validity of CVD risk prediction models [32]. Some studies have highlighted the fact that current risk predictor models produce skewed results in relation to ethnicity [28, 33].

Further issues in data collection were highlighted by the fact that Chinese patients made up 0.5% of the cumulative total of all the data collected in this review. While this is somewhat similar to the 0.7% recorded in the 2011 UK Census, three studies did not exclusively state the number of Chinese participants [17, 18, 21], and so was not available for analysis.

Bangladeshi, Indian and Pakistani ethnicities make up 5.6% of people recorded in the UK 2011 Census. The proportion grouped as South Asian ethnicity in this review was 5.1%. Black patients represent 3.1% of the patients in our review compared to 3.5% of the UK census. This suggests that patients are attending NHSHC almost proportionally to the UK population despite the overall low engagement with the service. It is important that we encourage Black and South Asian ethnicities to engage with the service as they are more likely to experience CVD [4, 5]. Therefore, the fact that there is an overall underrepresentation points to the need for effective interventions to engagement with patients regardless of ethnicity.

All studies suggested that patients from more affluent areas were more likely to attend appointments, be invited for appointments, and participate opportunistically compared to patients with higher levels of deprivation. The UK population trends show that deprivation interacts with ethnicity, as there is a higher percentage of ethnic minority communities living in the most deprived areas [34]. This finding supports the existing understanding of the inverse care law embedded in the UK healthcare system, whereby service users in more deprived areas struggle to access early interventions and so require more support with advanced diseases at diagnosis [35, 36]. This may explain the higher odds of CVD diagnosis in secondary care reported for ethnic minorities in the UK [5]. Improving access to this demographic would impact individual patient care, foster early diagnosis of treatable CVDs, and help minimise the NHS workforce challenges. Additionally, preventative interventions could lead to fewer hospital admissions of acute CVD, significantly reducing NHS costs in secondary care. Therefore, further investigation should be conducted on this topic.

### 4.2 Engagement with Services 

Our study shows a slight under-representation of the Black community attending NHSHCs, with 3.1% compared to 3.5% of the UK population (2011 census). This equates to 89% degree of representation, as shown in Table 2. This is concerning as we would expect a greater level of engagement with this community given the higher risk of developing risk factors of CVD [6]. Patel *et al*. [16] showed how the engagement with the black community increased when formal invitations to attend health checks were sent, suggesting that primary care providers could take a more proactive role in raising awareness and encouraging attendance rates for such services. However, Woringer *et al*. [22] found a greater representation of Black people attending health checks in other community settings (e.g., pharmacies, community centres, places of worship, libraries, and shopping centres) compared to the general population. In contrast to GP practices, these settings provide greater flexibility and are trusted due to their close connection with the community. As a result, training additional staff within these local settings could greatly enhance service attendance among minority communities.

We found that the South Asian group had the highest percentage (21.4–61.0%) of eligible patients attending health checks. However, the percentage of patients attending overall is low, which is important considering the increased CVD risk to this community [4]. These findings suggest that improvements are still needed to identify patients eligible for the service and encourage attendance, detecting risks earlier.

Participation rates among South Asians are significantly higher than those of the Chinese group at 11.6%–30.1% (do you mean 30% vs 11%), which may be related to cultural practices and ethnic differences in perceived CVD risk or awareness of the disease [37, 38]. Therefore, while more South Asian patients may be inclined to engage with the check and those of Chinese backgrounds may underestimate the benefits.

Our findings suggest that the number of eligible people attending the NHSHCs by ethnic groups could be higher, which in turn may improve awareness and maximise the benefit of the service. Certain patient groups considered to be eligible were not necessarily invited to attend the screening programs due to sociodemographic factors like homelessness or lack of registration at the local GP [39]. The latter point is important, particularly for new migrants, refugees or asylum seekers in the UK who may be unaware of the GP operation process and, therefore, miss the opportunity to participate in health protection schemes like the NHSHC. Evidence shows that some GPs express a reservation when registering patients without sufficient documentation despite guidelines encouraging more people to be seen at primary care [40, 41].

More opportunistic health checks were performed (n = 2966) compared to patients formally invited to the health checks (n = 2142). Notably, the odds of CVD risk were found to be higher among those assessed opportunistically [21], suggesting that individualised targeting of health checks to patients is an effective method in engaging with patients at risk of CVD.

Considering the trends in the NHSHC uptake [15, 16, 17, 18], there has been a steady increase in the number of patients attending appointments year on year. It is possible that as the new service was implemented with numerous public awareness campaigns, patients gradually found value in the programme, and so attendance improved accordingly. In 2013, Public Health implemented strategies to increase the number of eligible patients attending the NHSHC by delegating the responsibility of community engagement to local authorities [42]. This may have impacted the engagement of patients regionally and suggests reasoning for the disparities seen between local authorities in our study. Since data was collected for this review, there were plans to digitalise the service to make it more accessible to people via their own technology [43]. This would mean that patients could be assessed through their own devices at home. It is unclear whether this will further increase engagement; thus, future analysis should follow this.

Practices were financially incentivised at different rates, which could have impacted the implementation of the NHSHC scheme and the outreach of patients in the high-risk category, i.e. invitations to the service [18]. Additionally, Public Health England gave the responsibility of engagement to the Local Authorities to support NHSHC providers where needed. This regional difference in care could have impacted the quality of support patients were receiving.

The finding that South Asians and Black patients use NHSHC services more than the white group is counterintuitive, considering that the former groups reside in the most deprived areas associated with less engagement with screening initiatives. However, it is possible that the high risk of death from CVD in these groups may impact their awareness of the disease and, subsequently, participation in the scheme.

### 4.3 Inclusion of Ethnic Minority Communities 

Our finding suggests that patients accessing the service opportunistically had an increased risk of CVD by over 10% compared with those formally invited [21]. We cannot know whether these patients previously declined a formal invitation for the check or what prompted the GP to raise the issue during consultation. However, the number of people screened opportunistically was fewer than those formally invited to participate in the most deprived group. Again, this could be due to factors around registration at local GPs or other access-related issues, including distance from the GP or ability to take time off work [44]. In addition, across all eligible studies, the representation of this group is significantly lower than the more affluent groups. This lack of representation could suggest that patients are either not engaging with their GP or are not being offered the NHSHCs, even on an opportunistic basis. The fewer invitations to this demographic also suggests that there is less access to these services, which are designed to be available for all, as the NHS principles aim for [45].

Three studies highlight significant regional differences [18, 21, 22]. One study also highlighted that practices were financially incentivised at different rates, which could have impacted the implementation of the NHSHC scheme differently between regions of England and consequentially impacted the outreach of patients in the high-risk category [18].

Women were slightly over-represented in our review, suggesting they were more likely than men to attend the NHSHC screening program. It is reported that women use primary care more often [46], partly due to reproductive health. It is unclear whether this impacts on CVD screening. We are unaware of any UK study investigating gender differences by ethnicity in CVD risk screening. Ethnic minority women are at a greater risk of CVD risk factors [47]; therefore, exploring gender differences in CVD risk assessment may help identify where preventative treatment can be implemented.

## 5. Strengths and Limitations

Our study covered publications between 2012 and 2022, cumulatively sampling 6,622,374 NHSHCs, giving us a broad range of data to understand patient engagement with the service. We have addressed the socioeconomic differences of patients attending NHSHCs and explored potential reasons for this. This highlights that the services are inconsistent across regions of the UK. Where the existing literature focuses on the implementation and local management of the NHSHC, our review analyses the disparities in engagement. It offers recommendations for targeted intervention to improve outreach to minority communities.

Although this study aimed to be a comparative review of all ethnicities accessing services in the UK healthcare system, some ethnicities were not considered, as insufficient data was available. The category of Chinese patients was often pooled with ‘other Asian’ by the studies available to us, and therefore, this was classified as “other” for our study to ensure that the data collected for South Asian patients was not skewed. This was to ensure that the understanding of CVD being at greater risk to this community was not ignored. Additionally, we could not analyse the ethnic distribution of patients within the age, gender and deprivation categories, as data was not available.

Those without permanent home addresses are at a disadvantage in accessing NHS Health Check services by invitation. Thus, patients who are missing documentation may not have access to primary care services due to services requiring a registered home address and may, therefore, skew results. This includes newly migrated communities, refugees and patients experiencing homelessness. This is further limited by the fact that communities, such as travellers that access primary care, will be recorded as “White” despite additional socioeconomic barriers and educational barriers faced when accessing healthcare [48]. In addition to this, our review of deprivation was limited to the studies’ interpretation and whether they used Townsend or IMD scoring. Although both show similar trends across the UK [49], it is understood that there are differences found, particularly in urban areas. It was difficult to conclude similar findings across studies, especially those comparing urban areas, such as the London boroughs.

As all data points were collected from NHSHC collectively across the UK, assessing which local areas participated in each study was difficult. Therefore, some data collection points may overlap and over-represent some participants.

This review did not investigate the impact of CVD risk assessment on post-screening outcomes, including CVD-related incidents or mortality. We anticipate that future studies will provide more robust evidence on this aspect as the current Public Health England recommendation emphasises better collection of follow-up data to foster analysis and assessment of the impact on CVD outcomes [43].

## 6. Conclusions

This review found low engagement with the NHSHC service among patients. The lowest engagement is amongst Chinese and Black patients and the highest in South Asian patients. Engagement can be improved with a more proactive approach from GPs, inviting patients to attend and increasing awareness of the service amongst high-risk communities. Furthering this, the accessibility of appointments in the local community could also increase engagement with minority ethnic communities. Additional timeslots on weekends and evenings could encourage the use of preventative services. Our study found that a targeted approach, identifying patients eligible for the service and sending invitations, could increase engagement with patients at a higher risk. This would improve patient awareness of the service and improve outreach of the services to empower patients to attend. The fact that the overall engagement is low merits further intervention, which Public Health England recognises and is keen to improve.

Further research should include the differences in care experienced by women of colour as research suggests a data bias for women experiencing CVD, resulting in delays in help-seeking, diagnosis and treatment. With this additional barrier to health equality, it is important to explore the magnitude of delay and explore the reasons for this to support this marginalised community, who may not be aware of their risk and symptoms. Considering that 6.9% of NHSHCs have missing data on ethnicity, it is important to ensure accurate data collection of ethnicities in future studies to improve policies targeting specific groups at high risk.

## Data Availability

All data points generated or analyzed during this study are included in this article and there are no further underlying data necessary to reproduce the results.

## Abbreviations

CVD, Cardiovascular Disease; GP, General Practice; IMD, Index of Multiple Deprivation; NHS, National Health Service; NHSHC, National Health Service Health Check; PICOs, Population, Intervention, Context; PRISMA-DTA, Preferred Reporting Items for Systematic And Meta Analysis-Diagnostic Test Accuracy.

## Supplementary Material

Supplementary material associated with this article can be found, in the online version, at https://doi.org/10.31083/RCM25614.

## Appendix

See Appendix 1.

**Table 1.** **Detailing the search terms and use of search engines**.

Search engine	Search term
OVID (Medline)	1. Prevention.mp.
	2. Cardiovascular Disease.mp.
	3. ethnicity.mp
	4. primary care.mp.
	5. general practice.mp.
	6. community pharmacy.mp.
	7. family practice.mp.
	8. family doctor.mp.
	9. 4 or 5 or 6 or 7 or 8
	10. 1 and 2 and 3 and 9
	11. limit 10 to English language
	12. limit 11 to full text
	13. limit 12 to human
	14. limit 13 to humans
Web of Science	1. ALL = (primary care)
	2. ALL = (general practice)
	3. ALL = (community pharmacy)
	4. ALL = (family practice)
	5. ALL = (family doctor)
	6. ALL = (ethnicity)
	7. ALL = (Cardiovascular Disease)
	8. ALL = (prevention)
	9. #5 OR #4 OR #3 OR #2 OR #1
	10. #9 AND #8 AND #7 AND #6
PubMed	**Prevention AND Cardiovascular Disease AND Ethnicity AND (primary care OR General practice OR Community pharmacy OR Family practice OR “Family doctor”)** Filters: **Free full text, Full text** Sort by: **Most Recent**
	((“prevent”[All Fields] OR “preventability”[All Fields] OR “preventable”[All Fields] OR “preventative”[All Fields] OR “preventatively”[All Fields] OR “preventatives”[All Fields] OR “prevented”[All Fields] OR “preventing”[All Fields] OR “prevention and control”[MeSH Subheading] OR (“prevention”[All Fields] AND “control”[All Fields]) OR “prevention and control”[All Fields] OR “prevention”[All Fields] OR “prevention s”[All Fields] OR “preventions”[All Fields] OR “preventive”[All Fields] OR “preventively”[All Fields] OR “preventives”[All Fields] OR “prevents”[All Fields]) AND (“cardiovascular diseases”[MeSH Terms] OR (“cardiovascular”[All Fields] AND “diseases”[All Fields]) OR “cardiovascular diseases”[All Fields] OR (“cardiovascular”[All Fields] AND “disease”[All Fields]) OR “cardiovascular disease”[All Fields]) AND (“ethnical”[All Fields] OR “ethnically”[All Fields] OR “ethnicities”[All Fields] OR “ethnicity”[MeSH Terms] OR “ethnicity”[All Fields] OR “ethnic”[All Fields] OR “ethnics”[All Fields] OR “ethnology”[MeSH Subheading] OR “ethnology”[All Fields] OR “ethnology”[MeSH Terms]) AND (“primary health care”[MeSH Terms] OR (“primary”[All Fields] AND “health”[All Fields] AND “care”[All Fields]) OR “primary health care”[All Fields] OR (“primary”[All Fields] AND “care”[All Fields]) OR “primary care”[All Fields] OR (“general practice”[MeSH Terms] OR (“general”[All Fields] AND “practice”[All Fields]) OR “general practice”[All Fields]) OR (“pharmacies”[MeSH Terms] OR “pharmacies”[All Fields] OR (“community”[All Fields] AND “pharmacy”[All Fields]) OR “community pharmacy”[All Fields]) OR (“family practice”[MeSH Terms] OR (“family”[All Fields] AND “practice”[All Fields]) OR “family practice”[All Fields]) OR “Family doctor”[All Fields])) AND ((ffrft[Filter]) AND (fft[Filter]))
	**MeSH translation**
	**Prevention:** “prevent”[All Fields] OR “preventability”[All Fields] OR “preventable”[All Fields] OR “preventative”[All Fields] OR “preventatively”[All Fields] OR “preventatives”[All Fields] OR “prevented”[All Fields] OR “preventing”[All Fields] OR “prevention and control”[Subheading] OR (“prevention”[All Fields] AND “control”[All Fields]) OR “prevention and control”[All Fields] OR “prevention”[All Fields] OR “prevention’s”[All Fields] OR “preventions”[All Fields] OR “preventive”[All Fields] OR “preventively”[All Fields] OR “preventives”[All Fields] OR “prevents”[All Fields]
	**Cardiovascular Disease:** “cardiovascular diseases”[MeSH Terms] OR (“cardiovascular”[All Fields] AND “diseases”[All Fields]) OR “cardiovascular diseases”[All Fields] OR (“cardiovascular”[All Fields] AND “disease”[All Fields]) OR “cardiovascular disease”[All Fields]
	**Ethnicity:** “ethnical”[All Fields] OR “ethnically”[All Fields] OR “ethnicities”[All Fields] OR “ethnicity”[MeSH Terms] OR “ethnicity”[All Fields] OR “ethnic”[All Fields] OR “ethnics”[All Fields] OR “ethnology”[Subheading] OR “ethnology”[All Fields] OR “ethnology”[MeSH Terms]
	**Primary care:** “primary health care”[MeSH Terms] OR (“primary”[All Fields] AND “health”[All Fields] AND “care”[All Fields]) OR “primary health care”[All Fields] OR (“primary”[All Fields] AND “care”[All Fields]) OR “primary care”[All Fields]
	**General practice:** “general practice”[MeSH Terms] OR (“general”[All Fields] AND “practice”[All Fields]) OR “general practice”[All Fields]
	**Community pharmacy:** “pharmacies”[MeSH Terms] OR “pharmacies”[All Fields] OR (“community”[All Fields] AND “pharmacy”[All Fields]) OR “community pharmacy”[All Fields]
	**Family practice:** “family practice”[MeSH Terms] OR (“family”[All Fields] AND “practice”[All Fields]) OR “family practice”[All Fields]

MeSH terms were selected to be as inclusive as possible with our literature search, leaving more emphasis on excluding papers at review digression. mp, multi-purpose search.

Selection

1) Representativeness of the exposed cohort

a) Truly representative **(*one star*)**

b) Somewhat representative **(*one star*)**

c) Selected group

d) No description of the derivation of the cohort

2) Selection of the non-exposed cohort

a) Drawn from the same community as the exposed cohort **(*one star*)**

b) Drawn from a different source

c) No description of the derivation of the non-exposed cohort

3) Ascertainment of exposure

a) Secure record (e.g., surgical record) **(*one star*)**

b) Structured interview **(*one star*)**

c) Written self-report **(*one star*)**

d) No description

e) Other

Comparability

1) Comparability of cohorts based on the design or analysis controlled for confounders

a) The study controls for age, sex and marital status ***(one star)***

b) Study controls for other factors ***(one star)***

c) Cohorts are not comparable based on the design or analysis controlled for confounders

Outcome

1) Assessment of outcome

a) Independent blind assessment ***(one star)***

b) Record linkage ***(one star)***

c) Self-report

d) No description

e) Other

The maximum number of stars that can be awarded is 8.

6–8 stars is an excellent paper

4–6 stars is a good paper

2–4 stars is a satisfactory paper

Appendix 2: Newcastle Ottowa scale.

This tool assessed the quality of papers during the inclusion and exclusion criteria. The scale was adapted to our research [28]. This was used to evaluate the studies based on the representativeness of the cohort, the data collection methods, comparability, and outcome. Our review adapted the assessment of the outcome to negate stars awarded for follow-up studies, as this was not within the scope of our review. We also specified that controls for age, sex and marital status should be awarded a star as these would affect our primary aim of investigating ethnicity. Self-reported ascertainment of exposure was adapted to award a star as ethnicity is a self-reported risk factor and important to our findings. Demonstrating that the outcome was not present at the start of the study was also removed from our scale as all patients eligible for the NHSHC would have no previous history of cardiovascular disease.
